# Extrahepatic Bile Duct Resection in Gallbladder Carcinoma: Differentiated Discussion about Risk and Oncological Benefit

**DOI:** 10.4103/1319-3767.70630

**Published:** 2010-10

**Authors:** Ulrich Klaus Fetzner, Ionel Constantin Oana, Dirk L. Stippel

**Affiliations:** Department of General, Visceral and Cancer Surgery, University of Cologne, Kerpener-Strasse 62, DE-50937 Cologne, Germany. E-mail: ulrich.fetzner@uk-koeln.de; 1 Department of General and Visceral Surgery, Clinic Bad Hersfeld, Bad Hersfeld, Germany

Sir,

We read the article of Shukla and Barreto with interest.[1] This systematic review dealing with extrahepatic bile duct resection in surgical treatment of gallbladder carcinoma is unique and fundamental.

The basis of surgical strategy in solid cancer like gallbladder carcinoma must be the knowledge of local and disiminated spread of the cancer. Because of this, the background for actual recommendations of some authors of routine resection of the extrahepatic bile duct in gallbladder cancer appears completely unclear, particularly based on the well-known early and late complications of resection, anastomosis and reconstructions of the extrahepatic bile duct.

Miyazaki *et al*.[2] were the first who showed that direct infiltration of gallbladder carcinoma into the adjacent liver segments is the most common type of spread of this cancer. A macroscopic or microscopic extension in the direction of the ductus cysticus is rare. Also the simultaneous appearance in the extrahepatic bile duct is extremely seldom. Because of the high frequency of laparoscopic cholecystectomies, today gallbladder cancer is mostly accidentally diagnosed after an operation for a presumed benign disease.[3] In advanced gallbladder carcinoma, an extension into the direction of the cystic duct is associated with a high-graded bad prognosis.[2] This is mostly because of the limited chance of radical R0 resection under these situations.

The cited studies which advocate radical resection including resection of the extrahepatic bile duct mostly include even extended liver resection and an extended lymphadenectomy.[1] For this reason, it will scientifically be impossible to differentiate which procedure is responsible for the quoted survival benefit.

The importance of liver resection in surgical treatment of gallbladder carcinoma was recently – among many other authors – emphasized and proved by Araida *et al*. and Wakai *et al*.[4,5] Oncologic background is the importance of liverparenchyma in gallbladder carcinoma. The tumor infiltrates the liver directly and additionally micrometastasis can be displaced either hematogen-venous or by the retrograde lymphatic route, primarily in the functional right part of the liver.[2,4]

Yamaguchi *et al*.[6] found out that the thickness of liverparenchyma between the neck of the gallbladder and the right hepatic duct was only 1.6 mm (± 0.7 mm), so that the recommended safety distance of 2-3 cm can not be reached without anatomic liver resection, when the gallbladder carcinoma grows into the direction of the cystic duct. But to prevent later livermetastasis with the origin of hematogen or lymphatic tumor spread, even extended liver resections (hepatectomy, extended right hepatectomy) can be necessary from T2 stage on.[2] If this remains undone, local recurrence or livermetastasis impend, both the most common causes of death by gallbladder carcinoma.

The cited authors who advocate aggressive resection including resection of the extrahepatic bile duct are extraordinary experienced hepatobiliary surgeons. The results, especially the risks of these extended procedures cannot be compared and are not conferrable on a broad medical surgical supply level, and can therefore not be recommended generally.

The paper of Shukla and Barreto makes an important contribution to a differentiated and censorious consideration of the resection of the extrahepatic bile duct as part of the surgical strategy in operative treatment of gallbladder carcinoma. The final eight conclusions of Shukla and Barreto cover up exactly with our concept and our attitude to this subject.
